# Increased Aortic Arch Calcification and Cardiomegaly is Associated with Rapid Renal Progression and Increased Cardiovascular Mortality in Chronic Kidney Disease

**DOI:** 10.1038/s41598-019-41841-7

**Published:** 2019-03-29

**Authors:** Szu-Chia Chen, Melvin Teh, Jiun-Chi Huang, Pei-Yu Wu, Chiu-Yueh Chen, Yi-Chun Tsai, Yi-Wen Chiu, Jer-Ming Chang, Hung-Chun Chen

**Affiliations:** 1 Division of Nephrology, Department of Internal Medicine, Kaohsiung Medical University Hospital, Kaohsiung Medical University, Kaohsiung, Taiwan; 2 Faculty of Renal Care, College of Medicine, Kaohsiung Medical University, Kaohsiung, Taiwan; 3 School of Medicine, College of Medicine, Kaohsiung Medical University, Kaohsiung, Taiwan; 4 Department of Internal Medicine, Kaohsiung Municipal Hsiao-Kang Hospital, Kaohsiung Medical University, Kaohsiung, Taiwan; 5 Department of Nursing, Kaohsiung Municipal Hsiao-Kang Hospital, Kaohsiung, Taiwan; 6 Division of General Medicine, Department of Internal Medicine, Kaohsiung Medical University Hospital, Kaohsiung Medical University, Kaohsiung, Taiwan

## Abstract

Vascular calcification and cardiomegaly are highly prevalent in chronic kidney disease (CKD) patients. However, the association of the combination of aortic arch calcification (AoAC) and cardio-thoracic ratio (CTR) with clinical outcomes in patients with CKD is not well investigated. This study investigated whether the combination of AoAC and CTR is associated with poor clinical outcomes in CKD stages 3–5 patients. We enrolled 568 CKD patients, and AoAC and CTR were determined by chest radiography at enrollment. Rapid renal progression was defined as estimated glomerular filtration rate (eGFR) decline over 3 ml/min/1.73 m^2^ per year. Both AoAC score and CTR were significantly associated with rapid renal progression. High CTR was correlated with increased risk for cardiovascular mortality. We stratified the patients into four groups according to the median AoAC score of 4 and CTR of 50%. Those with AoAC ≥ 4 and CTR ≥ 50% (*vs*. AoAC score < 4 and CTR < 50%) were associated with eGFR decline over 3 ml/min/1.73 m^2^/year and cardiovascular mortality. AoAC and CTR were independently associated with eGFR slope. In conclusion, the combination of increased AoAC and cardiomegaly was associated with rapid renal progression and increased cardiovascular mortality in patients with CKD stage 3–5 patients. We suggest that evaluating AoAC and CTR on chest plain radiography may be a simple and inexpensive method for detecting CKD patients at high risk for adverse clinical outcomes.

## Introduction

Patients with chronic kidney disease (CKD) are at a high risk of end-stage renal disease (ESRD) and cardiovascular and all-cause mortality^1^. Vascular calcification (VC) in the aorta is considered to be a risk factor for cardiovascular morbidity and mortality in patients on long-term dialysis^1–4^. VC is commonly seen in patients with CKD or ESRD^5^. The process of VC is multifactorial and complex, and is associated with the metabolism of calcium and phosphorus, the loss of osteopontin and enhancing vascular osteoblasts and the proliferation of chondrocyte-like cells^4^. In addition, aging, diabetes, oxidative stress and inflammation have been shown to play roles in the pathophysiologic mechanisms of VC^4^.

Chest radiography is a non-invasive and inexpensive screening instrument to evaluate aortic arch calcification (AoAC) and cardiothoracic ratio (CTR). Accumulating evidence has shown an association between AoAC as measured using chest radiography and pulse pressure, intima-media thickness, and cardiovascular events in the general population^6,7^. Several recent studies have also reported that AoAC as assessed by chest radiography is associated with cardiovascular burden in patients with CKD or ESRD. In addition, Shin *et al*. reported a relationship between the severity of AoAC and brachial-ankle pulse wave velocity (baPWV), and that this could be a marker of arterial stiffness^8^. AoAC has also been shown to induce diastolic left ventricular dysfunction and left ventricular hypertrophy^4,8^. Moreover, AoAC has demonstrated to be a predictor of cardiovascular and all-cause mortality in patients with CKD or ESRD^4,9–12^. Furthermore, Li and colleagues reported a correlation between AoAC and poor renal function progression in patients with CKD not on dialysis^13^. Taken together, AoAC has an influence on adverse clinical outcomes in patients with CKD.

CTR is an easily obtained parameter related to left ventricular size, and a high CTR has been reported to be associated with elevated left ventricular mass and even left ventricular hypertrophy^14^. In patients on peritoneal dialysis, a high CTR has been associated with malnutrition-inflammation and fluid overload^15,16^, which then contribute to mortality and a rapid decline in renal function^13,16,17^. A positive correlation between AoAC and CTR has been shown in patients with CKD^13^, however whether CTR is correlated with adverse clinical outcomes and the impact of the combination of AoAC and CTR on adverse clinical outcomes are not clear in patients with CKD. Therefore, the aim of this study was to evaluate the relationship between a combination of AoAC and CTR and adverse clinical outcomes, including rapid renal function deterioration, cardiovascular mortality, and all-cause mortality in patients with CKD.

## Results

### Characteristics of the study patients

A total of 568 patients (343 men and 225 women) with CKD stages 3–5 were included. The mean age was 66.0 ± 12.2 years, and the median (IOR) AoAC score was 4 (3, 5). The mean percent error for AoAC measurements was 12.3 ± 12.3%. There was no significantly different AoAC score among CKD stages 3–5. 42.8% of the patients had a CTR ≥ 50%. The patients were stratified into four groups according to a median AoAC score of <4 or ≥4, and a CTR < 50% or ≥50%. Comparisons of the clinical characteristics among these study groups are shown in Table 1. There were 172, 109, 153 and 134 patients in the four groups, respectively. Compared to the patients in the AoAC < 4 and CTR < 50% group, those in the AoAC ≥ 4 and CTR ≥ 50% group were older, predominantly female, had a lower prevalence of smoking, higher prevalence of diabetes mellitus and hypertension, lower diastolic blood pressure, lower albumin, lower hemoglobin, lower baseline estimated glomerular filtration rate (eGFR), higher calcium-phosphorous product and higher urine protein-creatinine ratio (U_PCR_).

**Table 1:** Comparison of clinical characteristics among study groups.

Characteristics	AoAC ≤ 4 and CTR ≤ 50% (n = 172)	AoAC ≤ 4 and CTR > 50% (n = 109)	AoAC > 4 and CTR ≤ 50% (n = 153)	AoAC > 4 and CTR > 50% (n = 134)
Age (year)	62.1 ± 13.6	65.2 ± 12.8	67.5 ± 10.0^*^	69.7 ± 10.8^*†^
Male gender (%)	65.1	55.0	73.2^†^	44.0^*#^
Smoking (%)	32.2	26.6	35.9	21.6^#^
Diabetes mellitus (%)	47.1	56.9	60.9	67.9^*^
Hypertension (%)	80.8	89.9	82.3	91.8^*^
Coronary artery disease (%)	14.0	18.3	6.7^†^	18.7^#^
Cerebrovascular disease (%)	4.7	16.5^*^	12.0	11.2
Systolic blood pressure (mmHg)	142.3 ± 23.9	139.5 ± 20.4	145.7 ± 23.5	146.0 ± 23.0
Diastolic blood pressure (mmHg)	79.9 ± 12.7	76.7 ± 14.6	78.7 ± 14.6	73.8 ± 13.7^*#^
Body mass index (kg/m^2^)	25.3 ± 3.7	25.9 ± 4.0	25.0 ± 4.1	25.9 ± 3.8
**Laboratory parameters**				
Albumin (g/dL)	4.1 ± 0.4	4.0 ± 0.4^*^	4.1 ± 0.4	3.9 ± 0.4^*^
Fasting glucose (mg/dL)	122.5 ± 47.4	119.9 ± 49.9	136.8 ± 60.7	118.0 ± 51.3^#^
Triglyceride (mg/dL)	133.5 (94–189.5)	142 (93.5–200.5)	142.5 (102–202.25)	126.5 (84.75–189.5)
Total cholesterol (mg/dL)	198.4 ± 58.3	194.2 ± 55.6	198.1 ± 51.0	202.2 ± 51.6
HDL-cholesterol (mg/dL)	44.9 ± 12.9	43.8 ± 13.2	42.2 ± 13.0	46.2 ± 13.8
LDL-cholesterol (mg/dL)	112.3 ± 41.3	104.1 ± 39.1	114.2 ± 37.6	112.4 ± 40.1
Hemoglobin (g/dL)	11.7 ± 2.4	10.7 ± 2.2^*^	11.8 ± 2.0^†^	10.7 ± 2.1^*#^
Baseline eGFR (ml/min/1.73 m^2^)	28.6 ± 14.8	24.2 ± 12.5^*^	29.1 ± 14.1^†^	20.0 ± 12.0^*#^
Calcium-phosphorous product (mg^2^/dL^2^)	35.1 ± 6.4	37.6 ± 8.7	36.3 ± 7.3	40.1 ± 9.7^*#^
Upcr (mg/g)	1016.2 (414.6–2032)	1605 (512.5–3433.5)	989.3 (374.6–2500.5)	1975 (912.5–3894.325) ^*#^
**Medications**				
ACEI and/or ARB use	53.5	59.6	64.7	63.
**Outcome**				
eGFR slope (ml/min/1.73 m^2^/yr)	−1.67 ± 2.51	−2.74 ± 3.13	−3.05 ± 3.83^*^	−4.47 ± 4.05^*†#^
eGFR slope < −3 ml/min/1.73 m^2^/yr (%)	20.9	40.4^*^	38.6^*^	59.7^*†#^
All-cause mortality (%)	11.6	22.9	16.3	29.9^*#^
Cardiovascular mortality (%)	2.3	8.3	3.9	14.2^*#^

Abbreviations. AoAC, aortic arch calcification; CTR, cardiothoracic ratio; HDL, high-density lipoprotein; LDL, low-density lipoprotein; eGFR, estimated glomerular filtration rate; Upcr, Urine protein-to-creatinine ratio; ACEI, angiotensin converting enzyme inhibitor; ARB, angiotensin II receptor blocker.

The study patients were stratified into 4 groups according to median score of AoAC (4) and CTR ≤ 50% or > 50%.

^*^p < 0.05 com*p*ared AoAC ≤ 4 and CTR ≤ 50%; ^†^*p* < 0.05 compared with AoAC ≤ 4 and CTR > 50%; ^#^*p* < 0.05 compared with AoAC > 4 and CTR ≤ 50^%^.

Missing data number: body mass index (2), systolic blood press (3), diastolic blood pressure (3), hemoglobin (10), calcium-phosphorous product (25), albumin (13), cholesterol (14), triglyceride (25), high-density lipoprotein (24), low-density lipoprotein (23), fasting sugar (20), urine protein-to-creatinine ratio (30).

### Clinical outcomes among the study patients

Regarding outcomes, compared to the patients in the AoAC < 4 and CTR < 50% group, those in the AoAC ≥ 4 and CTR ≥ 50% group had a lower eGFR slope and higher prevalence of rapid renal progression with a decline in eGFR over 3 mL/min/1.73 m^2^/year, and higher all-cause and cardiovascular mortality (Table 1).

**Figure 1:**
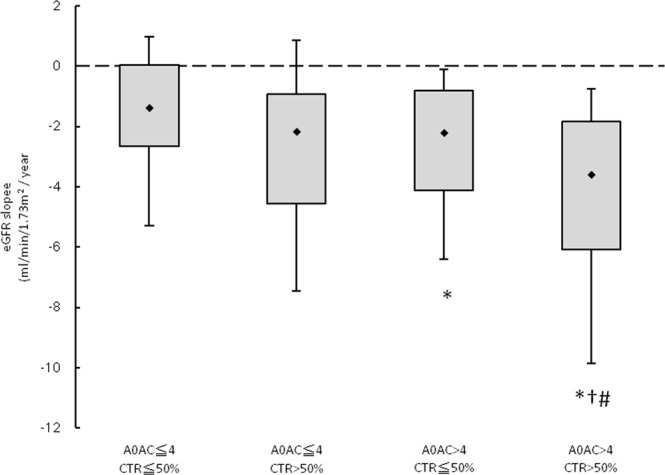
The estimated glomerular filtration rate (eGFR) slopes among 4 study groups. ^*^p < 0.05 compared aortic arch calcification (AoAC) ≤ 4 and CTR ≤ 50%; ^†^*p* < 0.05 compared with AoAC ≤ 4 and CTR > 50%; ^#^*p* < 0.05 compared with AoAC > 4 and CTR ≤ 50%.

Figure 1 illustrates that the eGFR slopes among the four study groups were −1.67 ± 2.54, −2.74 ± 3.13, −3.05 ± 3.83, and −4.47 ± 4.05 mL/min/1.73 m^2^/year, respectively. The eGFR slope was lowest in the AoAC ≥ 4 and CTR ≥ 50% group.

### AoAC and CTR and eGFR slope

Table 2 shows the determinants of the eGFR slope in all patients. In the univariate linear regression analysis, AoAC, CTR, diabetes mellitus, hypertension, diastolic blood pressure, fasting glucose, hemoglobin, baseline eGFR, calcium-phosphorous product, albumin, and log-formed UPCR were significantly correlated with eGFR slope. After multivariate analysis, we found that high AoAC, high CTR, young age, diabetes mellitus, low albumin, high U_PCR_, and not receiving angiotensin converting enzyme inhibitors (ACEI) and/or angiotensin II receptor blockers (ARB) were independently correlated with a larger negative eGFR slope.

**Table 2:** Determinants of estimated glomerular filtration rate (eGFR) slope using linear analysis in study patients.

Parameter	Univariate		Multivariate	
	Unstandardized coefficient β (95% CI)	*p*	Unstandardized coefficient β (95% CI)	*p*
AoAC (per 1 score)	−1.155(−1.468, 0.842)	<0.001	−1.008(−1.320, −0.697)	<0.001
CTR (per 1%)	−0.124(−0.174, −0.074)	<0.001	−0.061(−0.112, −0.010)	0.020
Age (per 1 year)	0.009(−0.015, 0.032)	0.483	0.045(0.020, 0.069)	<0.001
Gender (male *v.s*. female)	−0.180(−0.778, 0.418)	0.554	0.347(−0.245, 0.939)	0.251
Diabetes mellitus	−1.588(−2.167, −1.008)	<0.001	−0.672(−1.364, 0.019)	0.057
Hypertension	−1.737(−2.563, −0.911)	<0.001	−0.765(−1.611, 0.081)	0.076
Coronary artery disease	−0.474(−1.322, 0.373)	0.272		
Cerebrovascular disease	−0.111(−1.071, 0.849)	0.821		
ACEI and/or ARB use	0.338(−0.259, 0.935)	0.267	0.633(0.043, 1.224)	0.036
Systolic blood pressure (per 1 mmHg)	−0.012(−0.024, 0.001)	0.075		
Diastolic blood pressure (per 1 mmHg)	0.027(0.006, 0.047)	0.013	0.015(−0.007, 0.037)	0.174
Body mass index (per 1 kg/m^2^)	0.004(−0.071, 0.079)	0.917		
Fasting glucose (per 1 mg/dL)	−0.008(−0.013, −0.002)	0.007	−0.001(−0.007, 0.004)	0.583
Hemoglobin (per 1 g/dL)	0.319(0.192, 0.446)	<0.001	−0.055(−0.227, 0.117)	0.529
Baseline eGFR (per 1 mL/min/1.73 m^2^)	0.063(0.042, 0.083)	<0.001	0.020(00.008, 0.049)	0.163
Calcium-phosphorous product (per 1 mg^2^/dL^2^)	−0.048(−0.083, −0.012)	0.009	0.015(−0.022, 0.051)	0.435
Total cholesterol (per 1 mg/dL)	−0.003(−0.008, 0.003)	0.329		
Log-transformed triglyceride (log per 1 mg/dL)	−0.313(−1.545, 0.919)	0.618		
HDL-cholesterol (per 1 mg/dL)	−0.010(−0.033, 0.013)	0.377		
LDL-cholesterol (per 1 mg/dL)	−0.003(−0.010, 0.005)	0.445		
Albumin (per 1 g/dL)	2.766(2.100, 3.431)	<0.001	1.708(0.972,2.444)	<0.001
Upcr (log per 1 mg/g)	−2.315(−2.784, −1.845)	<0.001	−1.081(−1.688, 0.474)	0.001

Values expressed as unstandardized coefficient β and 95% confidence interval (CI). Abbreviations are the same as in Table 1.

Multivariate model: adjust for age, sex and ACEI/ARB usage plus variables of p-value < 0.05 in univariate analysis.

### AoAC, CTR and rapid renal progression

Logistic regression analysis was utilized to evaluate the association among AoAC, CTR and rapid renal progression (Table 3). In univariate analysis, AoAC, CTR, study group of AoAC ≥ 4 and CTR ≥ 50%, diabetes mellitus, hypertension, systolic blood pressure, fasting glucose, hemoglobin, baseline eGFR, calcium-phosphorous product, albumin, and log-formed UPCR were significantly correlated with rapid renal progression. In multivariate analysis model 1, high AoAC (odds ratio [OR], 2.264; 95% confidence interval [CI], 1.665 to 3.079; *p* < 0.001) and high CTR (OR, 1.059; 95% CI, 1.014 to 1.107; *p* = 0.010) were significantly correlated with rapid renal progression. Additionally, in model 2, compared to the AoAC < 4 and CTR < 50% group, the AoAC < 4 and CTR ≥ 50% (OR, 2.333; 95% CI, 1.225 to 4.440; *p* = 0.0.10), AoAC ≥ 4 and CTR < 50% (OR, 2.640; 95% CI, 1.404 to 4.966; *p* = 0.003) and AoAC ≥ 4 and CTR ≥ 50% (OR, 4.815; 95% CI, 2.474 to 9.369; *p* < 0.001) groups were significantly associated with rapid renal progression.

**Table 3:** Determinants of rapid renal progression (estimated glomerular filtration rate (eGFR) lope < −3 ml/min/1.73 m^2^/year) using logistic analysis in study patients.

Parameter	Univariate		Multivariate (model 1)		Multivariate (model 2)	
	OR (95% CI)	*p*	OR (95% CI)	*p*	OR (95% CI)	*p*
Study group						
AoAC < 4 and CTR < 50%	Reference		—	—	Reference	
AoAC < 4 and CTR ≥ 50%	2.557(1.505–4.346)	0.001	—	—	2.333(1.225–4.440)	0.010
AoAC ≥ 4 and CTR < 50%	2.371(1.451–3.874)	0.001	—	—	2.640(1.404–4.966)	0.003
AoAC ≥ 4 and CTR ≥ 50%	5.597(3.381–9.265)	<0.001	—	—	4.815(2.474–9.369)	<0.001
AoAC (per 1 score)	2.098(1.657–2.658)	<0.001	2.264(1.665–3.079)	<0.001	—	—
CTR (per 1%)	1.090(1.056–1.125)	<0.001	1.059(1.014–1.107)	0.010	—	—
Age (per 1 year)	0.991(0.978–1.005)	0.991	0.965(0.945–0.985)	0.001	0.972(0.952–0.991)	0.005
Gender (male *v.s*. female)	1.331(0.943–1.878)	0.103	0.815(0.495–1.341)	0.421	0.876(0.540–1.422)	0.592
Diabetes mellitus	2.112(1.482–3.010)	<0.001	1.225(0.709–2.118)	0.467	1.190(0.696–2.035)	0.525
Hypertension	2.687(1.528–4.725)	0.001	1.789(0.828–3.863)	0.139	1.810(0.850–3.856)	0.124
Coronary artery disease	1.565(0.970–2.525)	0.066	—	—	—	—
Cerebrovascular disease	0.724(0.408–1.287)	0.724	—	—	—	—
ACEI and/or ARB use	0.819(0.581–1.155)	0.255	0.836(0.502–1.390)	0.489	0.813(0.493–1.340)	0.416
Systolic blood pressure (per 1 mmHg)	1.009(1.002–1.017)	0.015	0.996(0.986–1.006)	0.383	0.998(0.988–1.008)	0.738
Diastolic blood pressure (per 1 mmHg)	0.991(0.979–1.003)	0.155	—	—	—	—
Body mass index (per 1 kg/m^2^)	1.009(0.967–1.054)	0.669	—	—	—	—
Fasting glucose (per 1 mg/dL)	1.005(1.002–1.008)	0.003	1.003(0.999–1.008)	0.172	1.004(0.999–1.008)	0.090
Hemoglobin (per 1 g/dL)	0.806(0.743–0.876)	<0.001	1.021(0.885–1.178)	0.779	1.039(0.902–1.197)	0.593
Baseline eGFR (per 1 mL/min/1.73 m^2^)	0.951(0.938–0.966)	<0.001	0.974(0.950–0.999)	0.044	0.973(0.950–0.997)	0.031
Calcium-phosphorous product (per 1 mg^2^/dL^2^)	1.040(1.017–1.063)	<0.001	0.990(0.960–1.021)	0.534	0.988(0.959–1.019)	0.454
Total cholesterol (per 1 mg/dL)	1.003(1.000–1.006)	0.078	—	—	—	—
Log-transformed triglyceride (log per 1 mg/dL)	1.584(0.770–3.258)	0.211	—	—	—	—
HDL-cholesterol (per 1 mg/dL)	1.009(0.996–1.023)	0.179	—	—	—	—
LDL-cholesterol (per 1 mg/dL)	1.001(0.997–1.006)	0.516	—	—	—	—
Albumin (per 1 g/dL)	0.184(0.112–0.305)	<0.001	0.398(0.208–0.763)	0.006	0.421(0.222–0.799)	0.008
Upcr (log per 1 mg/g)	5.920(3.884–9.023)	<0.001	2.849(1.618–5.018)	<0.001	2.931(1.684–5.102)	<0.001

Values expressed as odds ratio (OR) and 95% confidence interval (CI). Abbreviations are the same as in Table 1.

Multivariate model: adjust for age, sex and ACEI/ARB usage plus variables of p-value < 0.05 in univariate analysis.

**Figure 2:**
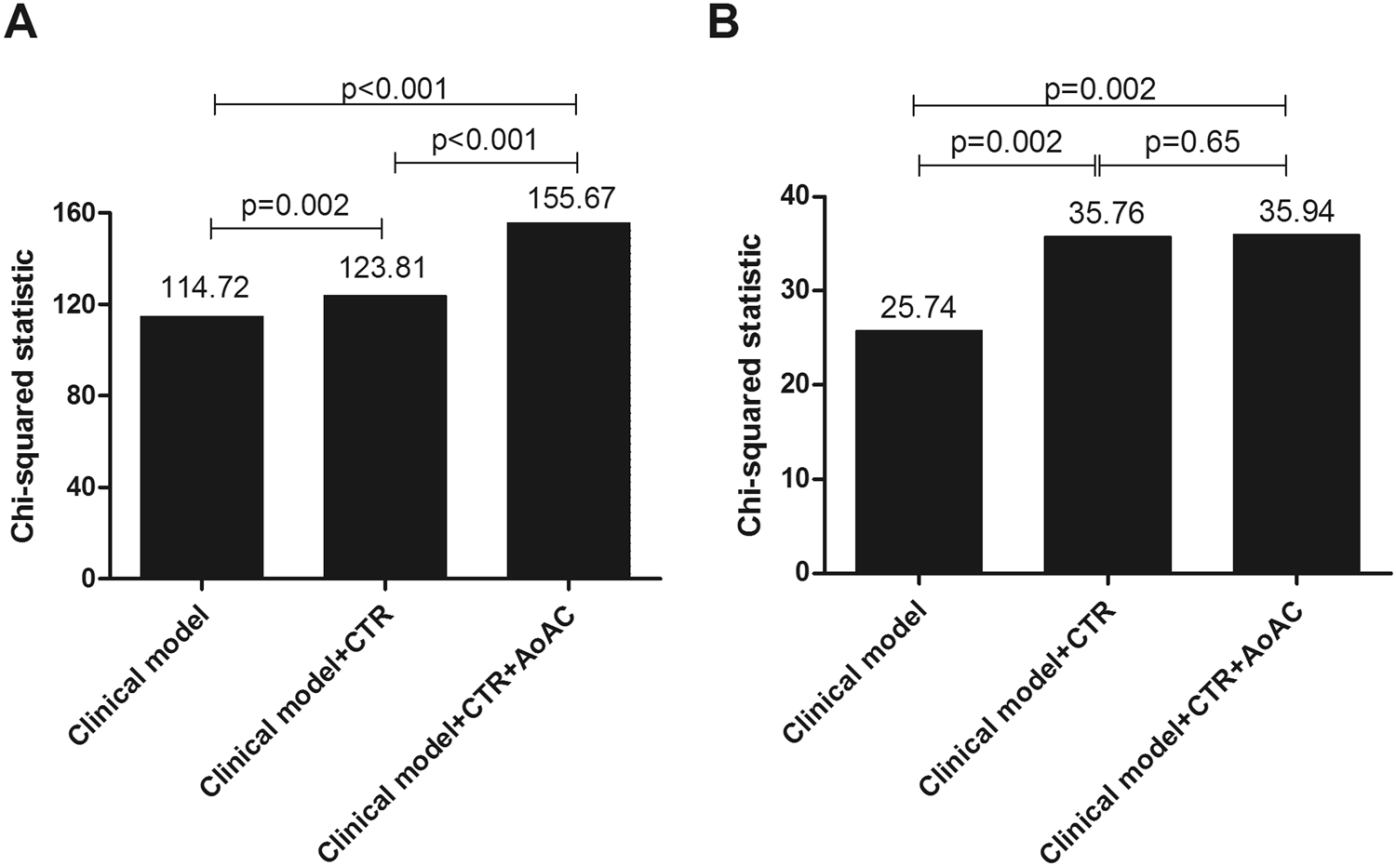
The assessment of predictive model of (**A**) rapid renal progression and (**B**) cardiovascular mortality. The clinical model included age, sex, and variables in Table 1 of p < 0.05 in univariate analysis.

Furthermore, we analyzed the effect of AoAC score and CTR on renal outcome prediction (Fig. 2A). The clinical model included age, sex, ACEI/ARB usage and variables with p < 0.05 in univariate analysis, including diabetes mellitus, hypertension, diastolic blood pressures, fasting glucose, hemoglobin, albumin, baseline eGFR, calcium-phosphorous product and proteinuria (χ^2^ = 114.72). Adding CTR to the clinical model offered an additional benefit in the prediction of rapid renal progression (χ^2^ = 123.81, p = 0.002). Besides, adding AoAC score to the model consisting of the clinical model and CTR resulted in further significant improvement in the prediction of rapid renal progression (χ^2^ = 155.67, p < 0.001).

### AoAC, CTR and all-cause mortality

**Figure 3:**
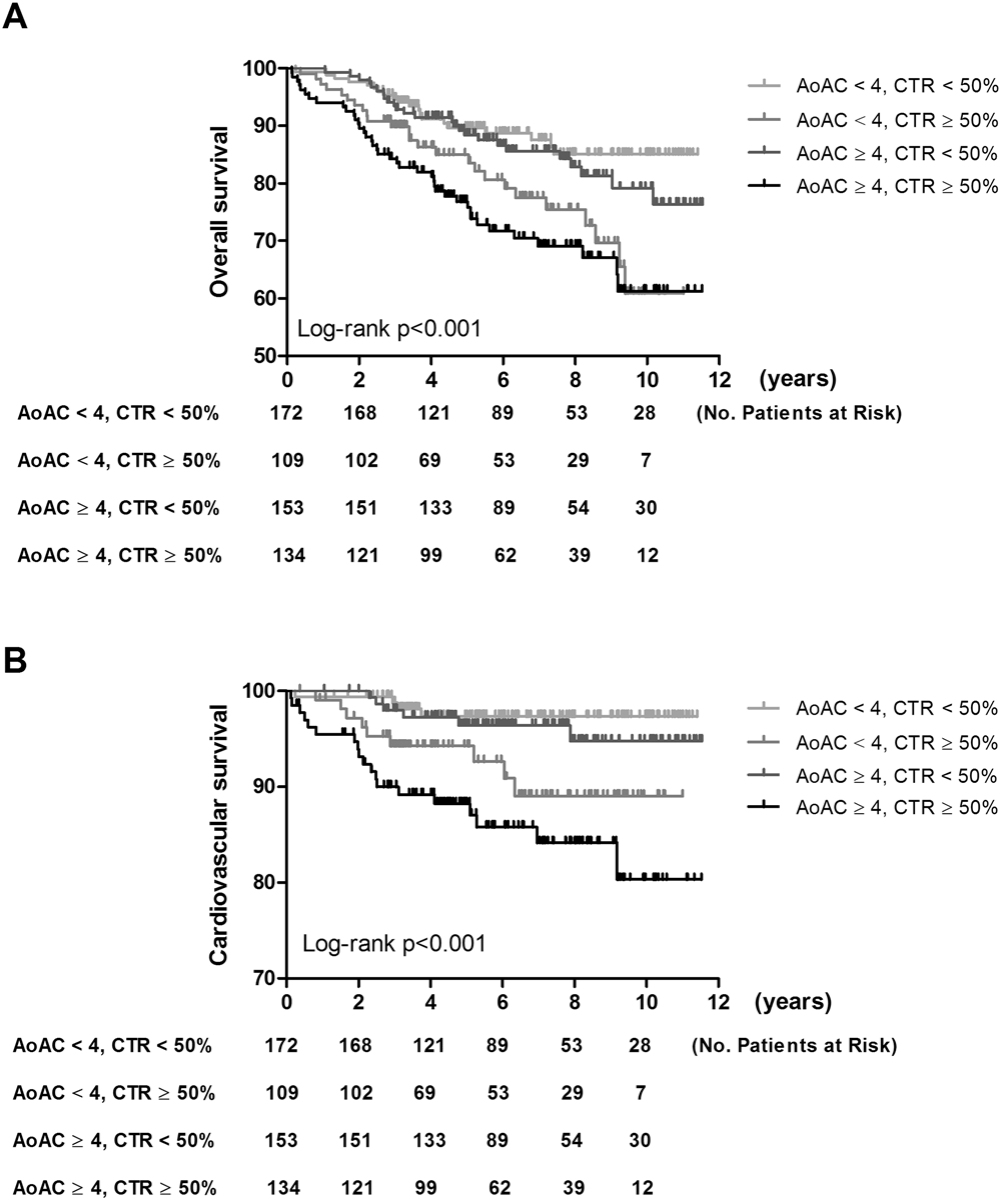
Kaplan-Meier analyses of (**A**) overall survival and (**B**) cardiovascular survival among 4 study groups. The group with AoAC ≤ 4 and CTR > 50% and the group with AoAC > 4 and CTR > 50% had worse overall survival than that with AoAC ≤ 4 and CTR ≤ 50%.

The mean follow-up period was 6.3 ± 2.8 years. During the follow-up period, 111 deaths were recorded among these 568 patients (19.5%), including cardiovascular deaths (n = 38), malignancy (n = 12), infectious diseases (n = 51), gastrointestinal bleeding (n = 4), and others (n = 6). Figure 3A illustrates the Kaplan-Meier analysis of overall survival (log-rank *p* < 0.001) among the four study groups. The AoAC < 4 and CTR ≥ 50% and AoAC ≥ 4 and CTR ≥ 50% groups had worse overall survival than the AoAC < 4 and CTR < 50% group. The AoAC ≥ 4 and CTR ≥ 50% group (*vs*. AoAC < 4 and CTR < 50% group) was associated with all-cause mortality in the unadjusted model (hazard ratio [HR], 2.800; 95% CI, 1.637 to 4.791; *p* < 0.001), however, this relationship was not significant after multivariate adjustments.

### AoAC, CTR and cardiovascular mortality

The causes of the 38 cardiovascular deaths during follow-up included heart failure (n = 10), myocardial infarction (n = 7), ventricular fibrillation (n = 19) and hemorrhagic stroke (n = 2). Figure 3B illustrates the Kaplan-Meier analysis of cardiovascular survival (log-rank *p* < 0.001) among the four study groups. The AoAC < 4 and CTR ≥ 50% and AoAC ≥ 4 and CTR ≥ 50% groups had worse cardiovascular survival than the AoAC < 4 and CTR < 50% group. Cox proportional hazards regression analysis of the association of AoAC and CTR with cardiovascular mortality is shown in Table 4. In univariate analysis, CTR, study group, body mass index, hemoglobin, and albumin were significantly associated with increased risk for cardiovascular mortality. In multivariate analysis, high CTR (HR, 1.100; 95% CI, 1.032 to 1.171; *p* = 0.003) were significantly associated with cardiovascular mortality, but AoAC was not. The AoAC ≥ 4 and CTR ≥ 50% group (*vs*. AoAC < 4 and CTR < 50% group) was associated with increased risk for cardiovascular mortality in the unadjusted model (HR, 6.624; 95% CI, 2.253 to 19.474; *p* = 0.001) and the relationship was still significant in multivariate adjusted model (HR, 4.127; 95% CI, 1.349 to 12.631; *p* = 0.013).

**Table 4:** Determinants of cardiovascular mortality using Cox proportional hazards model in study patients.

Parameter	Univariate		Multivariate (model 1)		Multivariate (model 2)	
	HR (95% CI)	*p*	HR (95% CI)	*p*	HR (95% CI)	*p*
Study group						
AoAC < 4 and CTR < 50%	Reference		—	—	Reference	
AoAC < 4 and CTR ≥ 50%	3.836(1.181–12.460)	0.025	—	—	3.242(0.991–10.609)	0.052
AoAC ≥ 4 and CTR < 50%	1.606(0.453–5.691)	0.463	—	—	1.302(0.365–4.650)	0.685
AoAC ≥ 4 and CTR ≥ 50%	6.624(2.253–19.474)	0.001	—	—	4.127(1.349–12.631)	0.013
AoAC (per 1 score)	1.502(0.959–2.353)	0.076	1.103(0.712–1.709)	0.661	—	—
CTR (per 1%)	1.113(1.057–1.173)	<0.001	1.100(1.032–1.171)	0.003	—	—
Age (per 1 year)	1.080(1.043–1.119)	<0.001	1.065(1.028–1.104)	<0.001	1.064(1.026–1.103)	0.001
Gender (male *v.s*. female)	1.806(0.955–3.416)	0.069	1.229(0.608–2.485)	0.566	1.261(0.623–2.551)	0.519
Diabetes mellitus	1.634(0.824–3.239)	0.160	—	—	—	—
Hypertension	6.637(0.911–48.382)	0.062	—	—	—	—
Coronary artery disease	1.821(0.834–3.975)	0.133	—	—	—	—
Cerebrovascular disease	1.658(0.693–3.967)	0.256	—	—	—	—
ACEI and/or ARB use	0.819(0.581–1.155)	0.255	—	—	—	—
Systolic blood pressure (per 1 mmHg)	1.001(0.987–1.015)	0.925	—	—	—	—
Diastolic blood pressure (per 1 mmHg)	0.984(0.961–1.008)	0.185	—	—	—	—
Body mass index (per 1 kg/m^2^)	0.909(0.831–0.996)	0.040	0.920(0.832–1.017)	0.104	0.918(0.830–1.014)	0.092
Fasting glucose (per 1 mg/dL)	1.001(0.995–1.007)	0.723	—	—	—	—
Hemoglobin (per 1 g/dL)	0.858(0.741–0.993)	0.040	1.006(0.832–1.215)	0.954	0.985(0.816–1.188)	0.872
Baseline eGFR (per 1 mL/min/1.73 m^2^)	0.976(0.951–1.001)	0.056	—	—	—	—
Calcium-phosphorous product (per 1 mg^2^/dL^2^)	1.008(0.971–1.047)	0.660	—	—	—	—
Total cholesterol (per 1 mg/dL)	0.998(0.992–1.005)	0.600	—	—	—	—
Log-transformed triglyceride (log per 1 mg/dL)	0.936(0.232–3.785)	0.926	—	—	—	—
HDL-cholesterol (per 1 mg/dL)	0.991(0.965–1.018)	0.524	—	—	—	—
LDL-cholesterol (per 1 mg/dL)	0.997(0.989–1.006)	0.515	—	—	—	—
Albumin (per 1 g/dL)	0.523(0.275–0.996)	0.049	0.607(0.270–1.365)	0.227	0.607(0.267–1.380)	0.233
Upcr (log per 1 mg/g)	1.000(1.000–1.000)	0.406	—	—	—	—

Values expressed as hazard ratio (HR) and 95% confidence interval (CI). Abbreviations are the same as in Table 1.

Multivariate model: adjust for age and sex plus variables of p-value < 0.05 in univariate analysis.

Besides, the effect of AoAC score and CTR on cardiovascular mortality prediction (Fig. 2B) was analyzed. The clinical model includes age, sex, and variables in Table 1 of p < 0.05 in univariate analysis (χ^2^ = 25.74). Adding CTR to the clinical model offered an additional benefit in the prediction of cardiovascular mortality (χ^2^ = 35.76, p = 0.002). However, adding AoAC score to the model consisting of the clinical model and CTR did not result in further significant improvement in the prediction of cardiovascular mortality in CKD patients.

### Sensitivity analysis

We used multiple imputation-expectation maximization to diminish the influence of missing data on the association among AoAC score, CTR, and clinical outcomes (Tables S1–3). The association of AoAC and CTR with rapid renal progression and cardiovascular mortality was still consistent.

## Discussion

This study investigated the association between the combination of AoAC and cardiomegaly with rapid renal function deterioration, overall and cardiovascular mortality in patients with CKD stages 3–5 over 6-years follow-up period. We found that both AoAC and cardiomegaly were significantly associated with rapid renal function deterioration with a decline in eGFR > 3 ml/min/1.73 m^2^/year, and that the coexistence of AoAC and cardiomegaly exacerbated the risk of rapid renal progression. In addition, the patients with CKD and both AoAC and cardiomegaly had a higher risk of cardiovascular mortality. The combination of AoAC and cardiomegaly could independently predict adverse clinical outcomes in the patients with CKD stages 3–5.

Many studies have shown that inflammation and oxidative stress are involved in the process of VC^18–20^. Inflammatory cytokines such as interleukin-6 and tumor necrosis factor have been shown to enhance vascular smooth muscle cell differentiation and VC^18,19^. Inflammation has also been shown to promote the production of reactive oxygen species, thereby further inducing vascular remodeling and the process of VC^19^. VC is associated with structural and functional alterations in the vessels, thereby disturbing the renal microcirculation^21^ and leading to impaired renal function. VC has also been shown to induce fibroelastic fiber thickening and a decrease in elastic fibers, and to cause elevated pulse pressure and arterial stiffness^22,23^. AoAC can augment cardiac afterload and arterial circumferential stress^22^, further promoting the onset of left ventricular hypertrophy^21^. These structural and functional changes to the cardiovascular system are associated not only with cardiovascular morbidity and mortality, but also with poor renal outcomes.

Accumulating evidence has shown that cardiomegaly is associated with survival in patients with ESRD^15–17^. Cardiomegaly is known to be correlated with malnutrition-inflammation status^16^, and it has also been reported to be a presentation of fluid overload^15^. Patients with cardiomegaly are susceptible to albuminuria, anemia, and fluid overload^15,16^. Fluid overload has been independently associated with rapid renal function deterioration and a high risk of cardiovascular burden^24–26^. To the best of our knowledge, the relationship between cardiomegaly and rapid renal progression remains unclear. In the current study, we found that both cardiomegaly and AoAC were positively correlated with rapid renal progression. Furthermore, the combination of AoAC and cardiomegaly could predict rapid renal progression in our patients with CKD. In addition, the coexistence of AoAC and cardiomegaly could predict cardiovascular mortality in these patients. The interaction between AoAC and cardiomegaly therefore appeared to have an effect on adverse clinical outcomes. These results indicate the application of a simple and useful tool, chest radiography, in predicting clinical outcomes to enhance CKD care.

Our results showed that the combination of AoAC and cardiomegaly was associated with all-cause mortality in an unadjusted model, but the significance was inconsistent after adjusting for demographic, clinical and biochemistry parameters. The relatively small number of enrolled patients and the small number who died may explain this finding. AoAC and cardiomegaly are cardiac indices, however, the cause of our death is mainly infection, which may also explain this finding. There were also other limitations to this study. AoAC and cardiomegaly were determinedusing plain radiography instead of computed tomography. Nevertheless, plain radiography is a simple and convenient tool to measure AoAC and cardiomegaly, and it is therefore easy for clinical physicians to monitor adverse clinical outcomes related to AoAC and cardiomegaly. Finally, AoAC and cardiomegaly were measured only once at enrollment. Therefore, the association between the effect of AoAC over time and cardiomegaly with clinical outcomes could not be estimated.

In conclusion, this study demonstrated that the combination of AoAC and cardiomegaly was significantly associated with rapid renal function deterioration and cardiovascular mortality in patients with CKD stages 3–5. Chest radiography is a widely available tool that can be used to evaluate AoAC and CTR, further providing clinical physicians with more information to detect patients with CKD at high risk of adverse clinical outcomes.

## Methods

### Study patients and design

**Figure 4:**
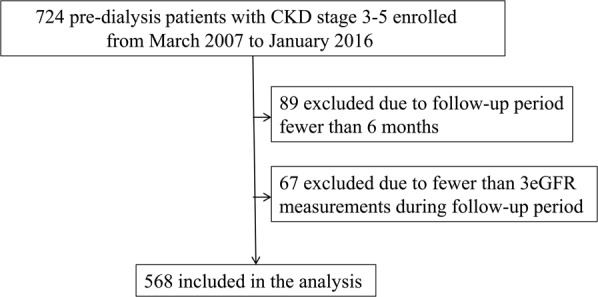
The flow chart of study patients enrollment.

This study was conducted at a regional hospital in southern Taiwan. We consecutively enrolled 724 pre-dialysis patients with CKD stages 3 to 5 according to the National Kidney Foundation-Kidney Disease Outcomes Quality Initiative (K/DOQI) guidelines^27^ from our Outpatient Department of Internal Medicine from March 2007 to January 2016. The eGFR was calculated using the equation of the 4-variable Modification of Diet in Renal Disease (MDRD) Study (eGFR = 186 × Serum creatinine^−1.154^ × Age ^−0.203^ × 0.742 (if female)^28^. The patients with evidence of kidney damage lasting >3 months based on an eGFR (mL/min/1.73 m^2^) of 30 to 59, 15 to 29, and <15 were defined as having CKD stages 3, 4, and 5, respectively. Sixty-seven patients had fewer than three eGFR measurements during the follow-up period, and they were excluded from this study. In addition, 89 patients who were followed up for <6 months were also excluded to avoid incomplete observations of changes in renal function. The remaining 568 patients (mean age 66.0 ± 12.2 years, 343 males) were included in this study (Fig. 4).

### Evaluation of AoAC and CTR by chest radiography

One experienced radiologist blinded to the patients’ clinical data reviewed the chest plain films of the enrolled patients with CKD. AoAC was evaluated using the scale system reported by Ogawa *et al*.^29^. The scale was calculated by dividing X-rays of the aortic arch into 16 sections by circumference, and then counting the number of sections with calcification. The reproducibility of AoAC data was assessed by an experienced radiologist and a medical doctor in 30 randomly selected patients. The mean percent error in each patient was calculated as absolute difference/average of two observations. As the report by Li and his colleague^13^, we defined the median of AoAC score (4.0) as cut-off value. AoAC score of 4 or above was regarded as high AoAC group.

CTR was defined as the ratio of the transverse diameter of the cardiac silhouette to the transverse diameter of the chest on the chest radiograph. Cardiomegaly was defined as a CTR > 50%.

### Collection of demographic, medical and laboratory data

Demographic and medical data including age, sex, smoking history (ever *vs*. never), and comorbidities were obtained from medical records and patient interviews. Body mass index was calculated as weight/square of the height (kg/m^2^). Fasting blood samples were obtained, and laboratory data were measured using an autoanalyzer (Roche Diagnostics GmbH, D-68298 Mannheim COBAS Integra 400). Serum creatinine was measured according to the compensated Jaffé (kinetic alkaline picrate) method using the same autoanalyzer (Roche/Integra 400, Roche Diagnostics) and a calibrator that could be used in isotope-dilution mass spectrometry^30^. EGFR was calculated using the four-variable Modification of Diet in Renal Disease (MDRD) equation^28^. Blood and urine samples were obtained within 1 month of enrollment. Data on the patients’ medications including ACEIs and ARBs during the study period were obtained from medical records.

### Clinical outcomes

Patients were contacted at outpatient clinics at 3-month intervals to ascertain the clinical status. Clinical outcomes included renal outcome, all-cause mortality and cardiovascular mortality. Renal outcome was defined as rapid renal progression. The eGFR slope was calculated by using regression coefficient between eGFR and time in units of ml/min per 1.73 m2 per year based on all eGFR values available from enrollment to the end of the observation period. The eGFR slope was only estimated when at least three eGFR measurements were available. A reduction of eGFR > 3 ml/min/1.73 m^2^/year was defined as rapid renal progression^31^. In the patients who received renal replacement therapy, renal function data were censored.

The information of all-cause and cardiovascular mortality was ascertained by reviewing medical records and the database of the National Mortality Files. The patients were followed until death or until September 2018.

### Statistical analysis

The study patients were stratified into four groups according to median values of AoAC (4) and CTR < or ≥50%. Data were expressed as percentages, mean ± standard deviation, or median (25^th^–75^th^ percentile) for triglycerides and U_PCR_. One-way analysis of variance followed by a Bonferroni post hoc test was used to compare variables among the study groups. Multiple linear regression analysis was used to identify the association of AoAC score and CTR with eGFR slope. Multiple logistic regression analysis was used to identify the association of AoAC score and CTR with rapid renal progression. Time-to-event survival analysis by Kaplan-Meier survival curve was used to test AoAC score or CTR as a predictor of the risk of overall and cardiovascular mortality. Cox proportional hazards model were utilized to evaluate the association of AoAC score and CTR with overall and cardiovascular mortality. The patients with AoAC < 4 and CTR < 50% were treated as the reference group, which was at the lowest risk of mortality. Incremental predictive model performance of clinical outcomes was assessed using a change in the χ^2^ value. Age, gender, and clinical variables those with p-value less than 0.05 in univariable analysis, were selected in multivariate analysis. We further did colinearity analysis in these adjusted variables (Table S4). A difference was considered significant at *p* < 0.05. Statistical analysis was performed using SPSS 19.0 for Windows (SPSS Inc. Chicago, USA).

### Statement of ethical approval

The study protocol was approved by the Institutional Review Board of Kaohsiung Medical University Hospital, and all of the patients provided written informed consent to participate in this study. The methods were carried out in accordance with the relevant guidelines.

## Supplementary information

Supplemental table

Supplementary Info File #1

## Data Availability

The datasets generated during and/or analysed during the current study are available from the corresponding author on reasonable request.
