# Supplementary material for: Increased Aortic Arch Calcification and Cardiomegaly is Associated with Rapid Renal Progression and Increased Cardiovascular Mortality in Chronic Kidney Disease
Source: Sci Rep. 2019 Mar 29;9:5354. doi: 10.1038/s41598-019-41841-7 (PMC6441024; doi:10.1038/s41598-019-41841-7)
Supplement: Supplementary file 1 — Supplemental table [file 41598_2019_41841_MOESM1_ESM.pdf]

Increased Aortic Arch Calcification and Cardiomegaly is Associated with Rapid Renal Progression and Increased Cardiovascular Mortality in Chronic Kidney Disease

Szu-Chia Chen, MD, PhD [1,2,3,4], Melvin Teh[3], Jiun-Chi Huang, MD[1,2,3,4], Pei-Yu Wu, MD[1,2,4], Chiu-Yueh Chen, RN[5], Yi-Chun Tsai, MD[1,2,3*], Yi-Wen Chiu, MD[1,2], Jer-Ming Chang, MD, PhD[1,2], Hung-Chun Chen, MD, PhD[1,2]

[1]Division of Nephrology, Department of Internal Medicine, Kaohsiung Medical University Hospital, Kaohsiung Medical University, Kaohsiung, Taiwan;

[2]Faculty of Renal Care, College of Medicine, Kaohsiung Medical University, Kaohsiung, Taiwan;

[3]School of Medicine, College of Medicine, Kaohsiung Medical University, Kaohsiung,

Taiwan;

[4] Department of Internal Medicine, Kaohsiung Municipal Hsiao-Kang Hospital, Kaohsiung Medical University, Kaohsiung, Taiwan

[5]Department of Nursing, Kaohsiung Municipal Hsiao-Kang Hospital, Kaohsiung, Taiwan

[*]Corresponding author

Yi-Chun Tsai, MD, PhD

Division of Nephrology, Department of Internal Medicine,

Kaohsiung Medical University Hospital,

Kaohsiung Medical University, Kaohsiung, Taiwan

100 TzYou 1st Road, Kaohsiung 807, TAIWAN

TEL: 886 7 3121101-5029   FAX: 886 7 3122810

E-mail: lidam65@yahoo.com.tw

Table S1. Determinants of estimated glomerular filtration rate (eGFR) slope using linear analysis in study patients after handling missing data by multiple imputation-expectation maximization

Parameter	Univariate		Multivariate	
	Unstandardized coefficient β (95% CI)	*p*	Unstandardized coefficient β (95% CI)	*p*
AoAC (per 1 score)	-1.155(-1.468, 0.842)	< 0.001	-0.997 (-1.301, -0.692)	<0.001
CTR (per 1%)	-0.124(-0.174, -0.074)	<0.001	-0.062(-0.112, -0.011)	0.016
Age (per 1 year)	0.009(-0.015, 0.032)	0.483	0.043(0.019, 0.067)	<0.001
Gender (male *v.s.* female)	-0.180(-0.778, 0.418)	0.554	0.350(-0.227, 0.928)	0.234
Diabetes mellitus	-1.588(-2.167, -1.008)	<0.001	-0.716 (-1.389, -0.044)	0.037
Hypertension	-1.737(-2.563, -0.911)	<0.001	-0.718 (-1.549, 0.113)	0.090
Coronary artery disease	-0.474(-1.322, 0.373)	0.272		
Cerebrovascular disease	-0.111(-1.071, 0.849)	0.821		
ACEI and/or ARB use	0.338(-0.259, 0.935)	0.267	0.599(0.026, 1.172)	0.041
Systolic blood pressure (per 1 mmHg)	-0.012(-0.024, 0.001)	0.074		
Diastolic blood pressure (per 1 mmHg)	0.027(0.006, 0.047)	0.013	0.014(-0.007, 0.035)	0.200
Body mass index (per 1 kg/m^2)	0.004(-0.071, 0.079)	0.913		
Fasting glucose (per 1 mg/dL)	-0.007(-0.013, -0.002)	0.010	-0.002(-0.007, 0.004)	0.541
Hemoglobin (per 1 g/dL)	0.312(0.183, 0.441)	<0.001	-0.062 (-0.230, 0.107)	0.471
Baseline eGFR (per 1 mL/min/1.73m^2)	0.063(0.042, 0.083)	<0.001	0.024(-0.005, 0.051)	0.085
Calcium-phosphorous product (per 1 mg^2/dL^2)	-0.049(-0.085, -0.013)	0.008	0.014(-0.022, 0.050)	0.446
Total cholesterol (per 1 mg/dL)	-0.003(-0.008, 0.003)	0.324		

Log-transformed triglyceride (log per 1 mg/dL)	-0.303(-1.534, 0.929)	0.629		
HDL-cholesterol (per 1 mg/dL)	-0.009(-0.032, 0.014)	0.454		
LDL-cholesterol (per 1 mg/dL)	-0.003(-0.010, 0.005)	0.486		
Albumin (per 1 g/dL)	2.747(2.068, 3.427)	< 0.001	1.725(1.012, 2.437)	<0.001
Upcr (log per 1 mg/g)	-2.228(-2.714, -1.742)	< 0.001	-1.051(-1.639, -0.463)	<0.001

Values expressed as unstandardized coefficient β and 95% confidence interval (CI). Abbreviations are the same as in Table 1.

Multivariate model: adjust for age, sex and ACEI/ARB usage plus variables of p-value <0.05 in univariate analysis

Table S2. Determinants of rapid renal progression (estimated glomerular filtration rate (eGFR) lope < -3 ml/min/1.73 m^2/year) using logistic analysis in study patients after handling missing data by multiple imputation - expectation maximization

Parameter	Univariate		Multivariate		Multivariate	
	OR (95% CI)	*p*	OR (95% CI)	*p*	OR (95% CI)	*p*
Study group						
AoAC < 4 and CTR < 50%	Reference		Reference		-	-
AoAC < 4 and CTR ≥ 50%	2.557(1.505-4.346)	0.001	2.299(1.249-4.234)	0.008	-	-
AoAC ≥ 4 and CTR < 50%	2.371(1.451-3.874)	0.001	3.273(1.813-5.911)	<0.001	-	-
AoAC ≥ 4 and CTR ≥ 50%	5.597(3.381-9.265)	<0.001	5.403(2.884-10.122)	<0.001	-	-
AoAC (per 1 score)	2.098(1.657-2.658)	<0.001	-	-	2.479(1.844-3.334)	<0.001
CTR (per 1%)	1.090(1.056-1.125)	<0.001	-	-	1.048(1.007-1.091)	0.021
Age (per 1 year)	0.991(0.978-1.005)	0.991	0.973(0.956-0.991)	0.004	0.967(0.948-0.986)	0.001
Gender (male *v.s.* female)	1.331(0.943-1.878)	0.103	0.864(0.551-1.354)	0.523	0.840(0.529-1.334)	0.461
Diabetes mellitus	2.112(1.482-3.010)	<0.001	1.107(0.679-1.805)	0.683	1.158(0.704-1.904)	0.565
Hypertension	2.687(1.528-4.725)	0.001	2.282(1.140-4.568)	0.020	2.198(1.090-4.432)	0.028
Coronary artery disease	1.565(0.970-2.525)	0.066	-	-	-	-
Cerebrovascular disease	0.724(0.408-1.287)	0.724	-	-	-	-
ACEI and/or ARB use	0.819(0.581-1.155)	0.255	0.719(0.450-1.148)	0.167	0.744(0.463-1.195)	0.221
Systolic blood pressure (per 1 mmHg)	1.009(1.002-1.017)	0.015	0.999(0.990-1.008)	0.827	0.996(0.987-1.006)	0.457
Diastolic blood pressure (per 1 mmHg)	0.991(0.979-1.003)	0.151	-	-	-	-
Body mass index (per 1 kg/m^2)	1.009(0.967-1.054)	0.672	-	-	-	-
Fasting glucose (per 1 mg/dL)	1.005(1.002-1.008)	0.004	1.005(1.000-1.009)	0.033	1.004(1.000-1.008)	0.080

Hemoglobin (per 1 g/dL)	0.807(0.743-0.876)	<0.001	1.017(0.892-1.159)	0.807	1.003(0.878-1.146)	0.961
Baseline eGFR (per 1 mL/min/1.73m^2)	0.951(0.938-0.966)	<0.001	0.970(0.949-0.992)	0.007	0.971(0.950-0.993)	0.011
Calcium-phosphorous product (per 1 mg^2/dL^2)	1.040(1.018-1.063)	<0.001	0.988(0.959-1.017)	0.417	0.992(0.963-1.022)	0.584
Total cholesterol (per 1 mg/dL)	1.003(1.000-1.006)	0.079	-	-	-	-
Log-transformed triglyceride (log per 1 mg/dL)	1.424(0.700-2.899)	0.330	-	-	-	-
HDL-cholesterol (per 1 mg/dL)	1.009(0.996-1.023)	0.181	-	-	-	-
LDL-cholesterol (per 1 mg/dL)	1.001(0.997-1.006)	0.522	-	-	-	-
Albumin (per 1 g/dL)	0.184(0.111-0.304)	<0.001	0.428(0.236-0.777)	0.005	0.409(0.223-0.750)	0.004
Upcr (log per 1 mg/g)	5.849(3.871-8.837)	<0.001	2.735(1.620-4.617)	<0.001	2.684(1.573-4.581)	<0.001

Values expressed as odds ratio (OR) and 95% confidence interval (CI). Abbreviations are the same as in Table 1.

Multivariate model: adjust for age, sex and ACEI/ARB usage plus variables of p-value <0.05 in univariate analysis

Table S3. Determinants of cardiovascular mortality using Cox proportional hazards model in study patients after handling missing data by multiple imputation - expectation maximization

Parameter	Univariate		Multivariate		Multivariate	
	HR (95% CI)	*p*	HR (95% CI)	*p*	HR (95% CI)	*p*
Study group						
AoAC < 4 and CTR < 50%	Reference		Reference		-	-
AoAC < 4 and CTR ≥ 50%	3.836(1.181-12.460)	0.025	3.214(0.981-10.529)	0.054	-	-
AoAC ≥ 4 and CTR < 50%	1.606(0.453-5.691)	0.463	1.311(0.368-4.673)	0.677	-	-
AoAC ≥ 4 and CTR ≥ 50%	6.624(2.253-19.474)	0.001	4.486(1.476-13.631)	0.008	-	-
AoAC (per 1 score)	1.502(0.959-2.353)	0.076	-	-	1.145(0.735-1.782)	0.550
CTR (per 1%)	1.113(1.057-1.173)	<0.001	-	-	1.100(1.034-1.171)	0.002
Age (per 1 year)	1.080(1.043-1.119)	<0.001	1.064(1.026-1.103)	0.001	1.066(1.029-1.104)	<0.001
Gender (male *v.s.* female)	1.806(0.955-3.416)	0.069	1.269(0.636-2.534)	0.500	1.224(0.615-2.438)	0.564
Diabetes mellitus	1.634(0.824-3.239)	0.160	-	-	-	-
Hypertension	6.637(0.911-48.382)	0.062	-	-	-	-
Coronary artery disease	1.821(0.834-3.975)	0.133	-	-	-	-
Cerebrovascular disease	1.658(0.693-3.967)	0.256	-	-	-	-
ACEI and/or ARB use	0.819(0.581-1.155)	0.255	-	-	-	-
Systolic blood pressure (per 1 mmHg)	1.001(0.987-1.015)	0.920	-	-	-	-
Diastolic blood pressure (per 1 mmHg)	0.984(0.961-1.008)	0.984	-	-	-	-
Body mass index (per 1 kg/m^2)	0.909(0.831-0.995)	0.039	0.915(0.829-1.011)	0.082	0.919(0.831-1.015)	0.096
Fasting glucose (per 1 mg/dL)	1.001(0.995-1.007)	0.743	-	-	-	-

Hemoglobin (per 1 g/dL)	0.864(0.747-0.999)	0.049	0.968(0.807-1.161)	0.729	0.986(0.821-1.183)	0.877
Baseline eGFR (per 1 mL/min/1.73m^2)	0.976(0.951-1.001)	0.056	-	-	-	-
Calcium-phosphorous product (per 1 mg^2/dL^2)	1.009(0.972-1.048)	0.632	-	-	-	-
Total cholesterol (per 1 mg/dL)	0.998(0.992-1.005)	0.624	-	-	-	-
Log-transformed triglyceride (log per 1 mg/dL)	0.908(0.234-3.528)	0.889	-	-	-	-
HDL-cholesterol (per 1 mg/dL)	0.991(0.965-1.018)	0.519	-	-	-	-
LDL-cholesterol (per 1 mg/dL)	0.997(0.989-1.006)	0.549	-	-	-	-
Albumin (per 1 g/dL)	0.528(0.277-1.005)	0.052	0.607(0.267-1.380)	0.233	0.607(0.270-1.365)	0.227
Upcr (log per 1 mg/g)	1.000(1.000-1.000)	0.340	-	-	-	-

Values expressed as hazard ratio (HR) and 95% confidence interval (CI). Abbreviations are the same as in Table 1.

Multivariate model: adjust for age and sex plus variables of p-value <0.05 in univariate analysis

Table S4. The colinearity analysis in variables of multivariate model

Parameter	Multivariate			
	Unstandardized coefficient β (95% CI)	*p*	Tolerance	VIF
AoAC (per 1 score)	-1.008(-1.320, -0.697)	<0.001	0.878	1.138
CTR (per 1%)	-0.061(-0.112, -0.010)	0.020	0.815	1.227
Age (per 1 year)	0.045(0.020, 0.069)	<0.001	0.786	1.272
Gender (male *v.s.* female)	0.347(-0.245, 0.939)	0.251	0.817	1.224
Diabetes mellitus	-0.672(-1.364, 0.019)	0.057	0.598	1.673
Hypertension	-0.765(-1.611, 0.081)	0.076	0.842	1.188
ACEI and/or ARB use	0.633(0.043, 1.224)	0.036	0.817	1.224
Diastolic blood pressure (per 1 mmHg)	0.015(-0.007, 0.037)	0.174	0.732	1.366
Fasting glucose (per 1 mg/dL)	-0.001(-0.007, 0.004)	0.583	0.815	1.227
Hemoglobin (per 1 g/dL)	-0.055(-0.227, 0.117)	0.529	0.463	2.158
Baseline eGFR (per 1 mL/min/1.73m^2)	0.020(00.008, 0.049)	0.163	0.468	2.137
Calcium-phosphorous product (per 1 mg^2/dL^2)	0.015(-0.022, 0.051)	0.435	0.720	1.389
Albumin (per 1 g/dL)	1.708(0.972,2.444)	<0.001	0.718	1.394
Upcr (log per 1 mg/g)	-1.081(-1.688, 0.474)	0.001	0.527	1.898
