# Supplementary material for: Increased Aortic Arch Calcification and Cardiomegaly is Associated with Rapid Renal Progression and Increased Cardiovascular Mortality in Chronic Kidney Disease
Source: Sci Rep. 2019 Mar 29;9:5354. doi: 10.1038/s41598-019-41841-7 (PMC6441024; doi:10.1038/s41598-019-41841-7)
Supplement: Supplementary file 2 — Supplementary Info File #1 [file 41598_2019_41841_MOESM2_ESM.pdf]

Increased Aortic Arch Calcification and Cardiomegaly is Associated with Rapid Renal Progression and Increased Cardiovascular Mortality in Chronic Kidney Disease

Szu-Chia Chen, MD, PhD [1,2,3,4], Melvin Teh[3], Jiun-Chi Huang, MD[1,2,3,4], Pei-Yu Wu, MD[1,2,4], Chiu-Yueh Chen, RN[5], Yi-Chun Tsai, MD[1,2,3*], Yi-Wen Chiu, MD[1,2], Jer-Ming Chang, MD, PhD[1,2], Hung-Chun Chen, MD, PhD[1,2]

[1]Division of Nephrology, Department of Internal Medicine, Kaohsiung Medical University Hospital, Kaohsiung Medical University, Kaohsiung, Taiwan;

[2]Faculty of Renal Care, College of Medicine, Kaohsiung Medical University, Kaohsiung, Taiwan;

[3]School of Medicine, College of Medicine, Kaohsiung Medical University, Kaohsiung, Taiwan;

[4] Department of Internal Medicine, Kaohsiung Municipal Hsiao-Kang Hospital, Kaohsiung Medical University, Kaohsiung, Taiwan

[5]Department of Nursing, Kaohsiung Municipal Hsiao-Kang Hospital, Kaohsiung, Taiwan

[*]Corresponding author

Yi-Chun Tsai, MD, PhD

Division of Nephrology, Department of Internal Medicine,

Kaohsiung Medical University Hospital,

Kaohsiung Medical University, Kaohsiung, Taiwan

100 TzYou 1st Road, Kaohsiung 807, TAIWAN

TEL: 886 7 3121101-5029   FAX: 886 7 3122810

E-mail: lidam65@yahoo.com.tw

STROBE statement-checklist of items that should be included in reports of observational studies.

	Item No	Recommendation	Checklist	Pages
**Title and abstract**	1	(*a*) Indicate the study's design with a commonly used term in the title or the abstract	Yes	1
		(*b*) Provide in the abstract an informative and balanced summary of what was done and what was found	Yes	3
**Introduction**				
Background/rationale	2	Explain the scientific background and rationale for the investigation being reported	Yes	5
Objectives	3	State specific objectives, including any prespecified hypotheses	Yes	6
**Methods**				
Study design	4	Present key elements of study design early in the paper	Yes	15
Setting	5	Describe the setting, locations, and relevant dates, including periods of recruitment, exposure, follow-up, and data collection	Yes	15
Participants	6	(*a*) *Cohort study*—Give the eligibility criteria, and the sources and methods of selection of participants. Describe methods of follow-up	Yes	15
		(*b*) *Cohort study*—For matched studies, give matching criteria and number of exposed and unexposed	N/A	
Variables	7	Clearly define all outcomes, exposures, predictors, potential confounders, and effect modifiers. Give diagnostic criteria, if applicable	Yes	15-16
Data sources/ measurement	8*	For each variable of interest, give sources of data and details of methods of assessment (measurement). Describe comparability of assessment methods if there is more than one group	Yes	16-17
Bias	9	Describe any efforts to address potential sources of bias	Yes	18
Study size	10	Explain how the study size was arrived at	Yes	15
Quantitative variables	11	Explain how quantitative variables were handled in the analyses. If applicable, describe which groupings were chosen and why	Yes	15-17
Statistical methods	12	(*a*) Describe all statistical methods, including those used to control for confounding	Yes	18
		(*b*) Describe any methods used to examine subgroups and interactions	Yes	18
		(*c*) Explain how missing data were addressed	Yes	18
		(*d*) *Cohort study*—If applicable, explain how loss to follow-up was addressed	Yes	17
		(*e*) Describe any sensitivity analyses	Yes	18
**Results**				
Participants	13*	(a) Report numbers of individuals at each stage of study—eg numbers potentially	Yes	7,15

		eligible, examined for eligibility, confirmed eligible, included in the study, completing follow-up, and analyzed		
		(b) Give reasons for non-participation at each stage	Yes	15
		(c) Consider use of a flow diagram	Yes	Figure 4
Descriptive data	14*	(a) Give characteristics of study participants (eg demographic, clinical, social) and information on exposures and potential confounders	Yes	7, Table 1
		(b) Indicate number of participants with missing data for each variable of interest	Yes	Table 1
		(c) *Cohort study*—Summarise follow-up time (eg, average and total amount)	Yes	10
Outcome data	15*	*Cohort study*—Report numbers of outcome events or summary measures over time	Yes	10, Table 1
		Case-control study—Report numbers in each exposure category, or summary measures of exposure	N/A	
		Cross-sectional study—Report numbers of outcome events or summary measures	N/A	
Main results	16	(*a*) Give unadjusted estimates and, if applicable, confounder-adjusted estimates and their precision (eg, 95% confidence interval). Make clear which confounders were adjusted for and why they were included	Yes	8-11, Table 2,3,4
		(*b*) Report category boundaries when continuous variables were categorized	Yes	8-11, Table 2,3,4
		(*c*) If relevant, consider translating estimates of relative risk into absolute risk for a meaningful time period	N/A	
Other analyses	17	Report other analyses done—eg analyses of subgroups and interactions, and sensitivity analyses	Yes	11
Discussion				
Key results	18	Summarize key results with reference to study objectives	Yes	12
Limitations	19	Discuss limitations of the study, taking into account sources of potential bias or imprecision. Discuss both direction and magnitude of any potential bias	Yes	14
Interpretation	20	Give a cautious overall interpretation of results considering objectives, limitations, multiplicity of analyses, results from similar studies, and other relevant evidence	Yes	12-14
Generalisability	21	Discuss the generalisability (external validity) of the study results	Yes	13-14
**Other information**				
Funding	22	Give the source of funding and the role of the funders for the present study and, if applicable, for the original study on which the present article is based	Yes	25
